# Crowning a Badly Decayed Root

**Published:** 1904-06

**Authors:** F. J. Blight

**Affiliations:** Liverpool, Eng.


### CROWNING A BADLY DECAYED ROOT.

By F. J. Blight, Liverpool, Eng.

Some time ago I was asked by a patient if I would crown an upper central incisor, which had been filled some years back with a very large gold filling, and which had unfortunately given way. On examination I found that the decay had spread largely into the root, involving considerably the pulp canal; after carefully excavating all decay and *debris,* nothing remained but little more than a shell of the original root. My patient still desired me to crown because of his dislike to a denture.

Well, gentlemen, to be brief: (1) I took a piece of platinum foil, of about the thickness of No. 1, and rolled it into the shape of a cone. This I placed into the root and packed the foil gently against the sides with cotton wool (amadou may be used) and so obtained an exact shape or impression of the root. (2) I removed the cotton wool and filled in the hollow cone with wax (whilst still in the root). (3) I then invested this in sand and plaster and filled in with scrap gold, and when the cone was almost filled I placed a small platinum pin in for manipulation. (4) The now solid cone was placed into the root, and a piece of platinum foil No. 2 was carefully burnished over the edges of the root for a base plate and soldered, and preceeded as with an ordinary pivot.

The building up of a post for a root (as above described) is to my mind an improvement over the older methods, viz., either undercutting and packing in with amalgam, or narrowing the root canal by cementing in a tube taken from a broken tube tooth.

If pin wire be introduced into the solid cone, a tube tooth may very easily be let down; or if pure gold be used throughout in solderng, a tooth may be fused with either Ash's or Jenkin's body.—*Jour. B. D. A.*

